# DGS-YOLO: A Detection Network for Rapid Pig Face Recognition

**DOI:** 10.3390/ani16020187

**Published:** 2026-01-08

**Authors:** Hongli Chao, Wenshuang Tu, Tonghe Liu, Hang Zhu, Jinghuan Hu, Tianli Hu, Yu Sun, Ye Mu, Juanjuan Fan, He Gong

**Affiliations:** 1 College of Information Technology, Jilin Agricultural University, Changchun 130118, China; chaohongli@mails.jlau.edu.cn (H.C.); tuyouyou050630@mails.jlau.edu.cn (W.T.); zhuhang@mails.jlau.edu.cn (H.Z.); 20231337@mails.jlau.edu.cn (J.H.); hutianli@jlau.edu.cn (T.H.); sunyu@jlau.edu.cn (Y.S.); muye@jlau.edu.cn (Y.M.); 2 Jilin Provincial Agricultural Mechanization Management Center, Changchun 130118, China; liutonghe970928@foxmail.com; 3 Jilin Province Agricultural Internet of Things Technology Collaborative Innovation Center, Changchun 130118, China; 4 Jilin Province Intelligent Environmental Engineering Research Center, Changchun 130118, China; 5 School of Electronic Science and Engineering, Jilin University, Changchun 130015, China

**Keywords:** facial recognition, YOLOv11, feature extraction

## Abstract

This study addresses the practical demand for facial recognition of pigs in the food safety and insurance industries, tackling the challenge of low recognition accuracy caused by complex farming environments, occlusions, and similar textures. To this end, we propose an enhanced model, DGS-YOLO, based on YOLOv11n, designed to achieve precise facial recognition of group-raised young pigs. The core improvements of the model include the following: (1) replacing standard convolutions with dynamic convolutions (DMConv) to enhance the network’s adaptive extraction capability for critical detail features; (2) designing a C3k2_GBC module with a bottleneck structure to replace the C3k2 neck, enabling more efficient capture of multi-scale contextual information; (3) introducing the SimAM parameter-free attention mechanism to optimize feature focusing; (4) employing the Shape-IoU loss function to mitigate the impact of bounding box geometry on regression accuracy. Experiments on self-built datasets demonstrate that DGS-YOLO achieves 4%, 2.1%, and 2.3% improvements in accuracy, recall, and mAP50, respectively, compared to the baseline model YOLOv11n. Furthermore, its overall performance surpasses that of Faster R-CNN and SSD in comprehensive evaluation metrics. Especially in limited sample scenarios, the model demonstrates strong generalization ability, with accuracy and mAP50 further increased by 20.1% and 10.3%. This study provides a highly accurate and robust solution for animal facial recognition in complex scenarios.

## Simple Summary

To address the challenge of insufficient facial recognition accuracy in animal protection, this paper proposes DGS-YOLO—a pig face recognition model based on an improved YOLOv11n—using pigs in group housing facilities as the research subject. The model focuses on optimizing recognition performance under interfering factors such as complex backgrounds, subtle textures, and facial occlusions, effectively enhancing detection accuracy and robustness. Experimental results demonstrate that DGS-YOLO achieves a precision of 88.3%, a recall rate of 86.9%, and an mAP50 of 91.8% in pig face recognition tasks.

## 1. Introduction

With the large-scale development of pig farms, there remain certain shortcomings in disease prevention and control. As the insurance industry enters the field, facial recognition technology for pigs has garnered widespread attention. Precision Livestock Farming (PLF) utilizes modern information technology to enable real-time monitoring of individual livestock and poultry, thereby achieving precise management and optimizing their production performance. At present, most applications for animal identification involve ear tags, collars, temperature sensors, and radio frequency identification (RFID) sensors. The use of ear tags can cause physical damage to the animal, leading to symptoms such as fever and inflammation. Pigs may damage collars and temperature sensors by biting each other, and these devices fade over time. Replacing them is time-consuming and labor-intensive, leading to economic losses and making large-scale pen rearing impractical. RFID requires pigs to actively approach readers for identification, and personnel cannot visually distinguish individual pigs. Pigs possess distinct facial patterns, eyes, and noses, yet visual identification remains challenging. This paper proposes a deep learning-based facial recognition system for pigs that avoids physical harm to the animals and prevents economic losses.

Deep learning-based computer vision technology possesses highly efficient autonomous feature learning capabilities and continuous evolutionary potential, enabling a wide range of applications in detection, classification, recognition, and tracking in the new phase of development. Mainstream algorithms primarily include two-stage detection algorithms and single-stage detection algorithms. Among the two-stage detection algorithms, R-CNN [1] and Faster R-CNN [2] are the most commonly used. However, their complex model structures result in slow training and inference speeds, low accuracy for detecting extremely small or large objects, excessive memory consumption, and deployment challenges. Another class of stage-based detection algorithms bypasses the region proposal step, feeding input images directly to the neural network. Key examples include SSD [3] and the YOLO series. SSD exhibits strong dependence on hyperparameters for object detection, resulting in high tuning costs. It performs independent predictions on multi-scale feature maps, lacking information exchange. YOLO achieves end-to-end training with more efficient backbone and neck networks, along with advanced label allocation and training strategies. Its low model complexity and high computational efficiency make it the preferred choice for deployment on embedded devices. YOLO strikes an excellent balance between accuracy, speed, and efficiency in complex scenes.

There are also numerous experiments on facial recognition in animals. For example, Yan [4] and others used traditional machine learning models to study the impact of LDA preprocessing on pig face recognition using SVM, employing both SVM models and models combining LDA with SVM, with core functions being polynomial and RBF, deployed in SVM classifiers on mobile and embedded systems. Luo et al. [5] employed models such as VGG, GoogLeNet, ResNet, MobileNet, and ConvNeXt to conduct cross-temporal training on pig faces. Within the VGG model, ReLU activation functions were used to connect each convolutional layer, and facial recognition was performed on data collected over a 49-day period. Liu et al. [6] proposed the RC2f module to replace the original C2f module by drawing inspiration from the Res2Net network architecture. They incorporated the ECA attention mechanism and Hardswish activation function into the Conv module, merged the independent Detect branch into the PFDetect detector head, and established the PFL-YOLO model for sheep face recognition. This overall model reduced the number of parameters and computational complexity, thereby improving the operational efficiency of the entire model. Gao et al. [7] employed YOLOv7 for bovine face recognition. They first integrated RFB and CBF modules into the backbone network, applied CBAM feature fusion in the neck network, and finally optimized the model using the SIoU loss function, thereby further enhancing detection accuracy and robustness. Chen [8] employed the ResNet50 model for facial recognition in Holstein cattle. Building upon the GPN model, they integrated the STN module with an attention mechanism to construct the GPN-ST model, which extracts local features to enhance recognition performance. Weng et al. [9] extended the oriented FAST and rotated BRIEF algorithms for bovine face detection, employing a dual-branch matching algorithm that combines the ORB algorithm with the GMS algorithm to enhance the accuracy of bovine face recognition.

Most of the aforementioned methods are applied to individual subjects or animals with easily distinguishable facial features. However, in group-housed pig environments, issues such as external objects obstructing facial views, individual pigs being obscured by others, and incomplete extraction of facial detail features when equipment is placed at a distance make precise facial recognition impractical. To address the above issues, this paper proposes a DGS-YOLO model for facial recognition of pigs during activity. Building upon YOLOv11n, it enhances accurate target identification and improves extraction of detailed features, making it suitable for group housing pig barns. The main contributions of this paper are as follows:

(1) We construct an indoor pig facial dataset covering faces at different times, angles, and with various occlusions. This dataset is used to train and evaluate all models presented in this paper. Addressing current challenges in facial feature detection, we propose the DGS-YOLO model based on YOLOv11n, which incorporates occlusion and texture detail features.
(2) We propose the DMConv module, which combines the Mish function with the DynamicTanh module to enhance texture detail features while suppressing noise interference.
(3) Using the C3k2-GBC module, features are mapped from high-dimensional to low-dimensional space, effectively capturing multi-scale information for precise target localization. Dynamic weighting adjustments for complex backgrounds and multi-morphological features reduce the impact of dirt and other obstructions on facial recognition. This achieves interaction and enhancement of detailed features.
(4) Adding the SimAM attention mechanism before the detection head enables a final optimization of fine-grained features, achieving optimal accuracy.
(5) We replace the original loss function with Shape-IoU to mitigate the impact of the inherent scale and shape of object boundaries on accuracy.

## 2. Materials and Methods

### 2.1. Data Collection and Processing

**Figure 1:**
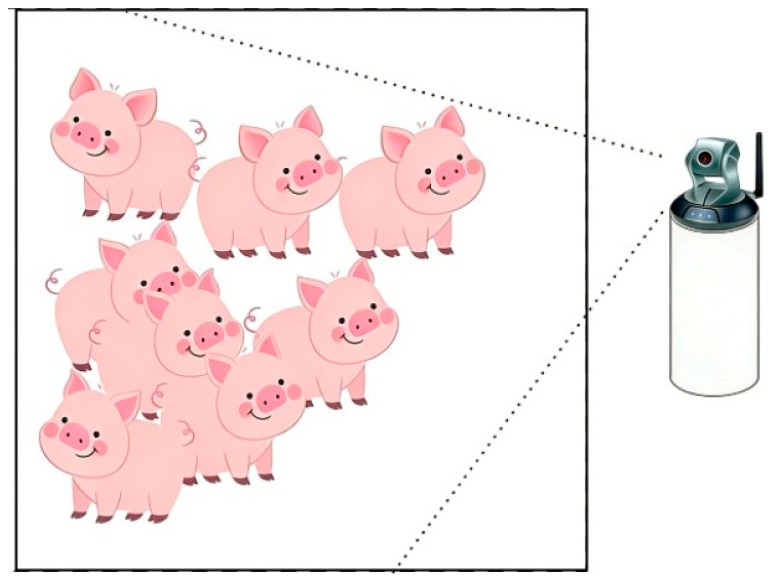
Camera mounting position.

The experimental data collection for this study was completed during the summer at a standardized breeding farm in Gongzhuling City, Jilin Province. To ensure that the captured pig facial images adequately encompassed varying lighting conditions and multiple occlusion scenarios, the entire imaging period lasted 7 days. Daily collection sessions were scheduled from 8:30 a.m. to 5:30 p.m. to capture diverse natural light variations from morning through evening. The pig pens were uniformly configured as enclosed 4 m × 4 m areas, each housing eight three-month-old piglets to simulate realistic group interactions under moderate stocking density conditions. In a completely natural rearing environment, we employed a network camera with a resolution of 1920 × 1080 and a video frame rate of 30 fps for continuous recording, focusing on capturing the facial features and behavioral dynamics of piglets. The dataset constructed through systematic collection comprehensively encompasses various complex scenarios commonly encountered in pig barns, including fluctuations in light intensity, partial facial occlusion, motion blur, diverse shooting angles, and the coexistence of single and multiple targets. It effectively reflects the data diversity inherent in real-world farming environments. The original dataset comprises 12 video clips and 921 static photographs. To enhance image diversity and reduce data redundancy, we extracted frames from the video material at a rate of one frame per second. Subsequently, we rigorously screened the extracted images based on visual similarity and facial completeness criteria, eliminating those with excessive redundancy, missing faces, or unrecognizable features. Visual color coding was employed to effectively distinguish and label eight independent individuals (ID: 0, 1, 2, 3, 4, 5, 7, 10). This process yielded a total of 4646 pig facial images, constituting the experimental dataset for subsequent training and validation of the pig facial recognition model. All images were annotated with bounding boxes using the Labelimg tool and randomly divided into training and validation sets at an 8:2 ratio. During data collection, the camera equipment remained fixed at a designated position on one side of the pig pen, with the specific installation method shown in Figure 1.

### 2.2. Method

#### 2.2.1. YOLO Improvements

**Figure 2:**
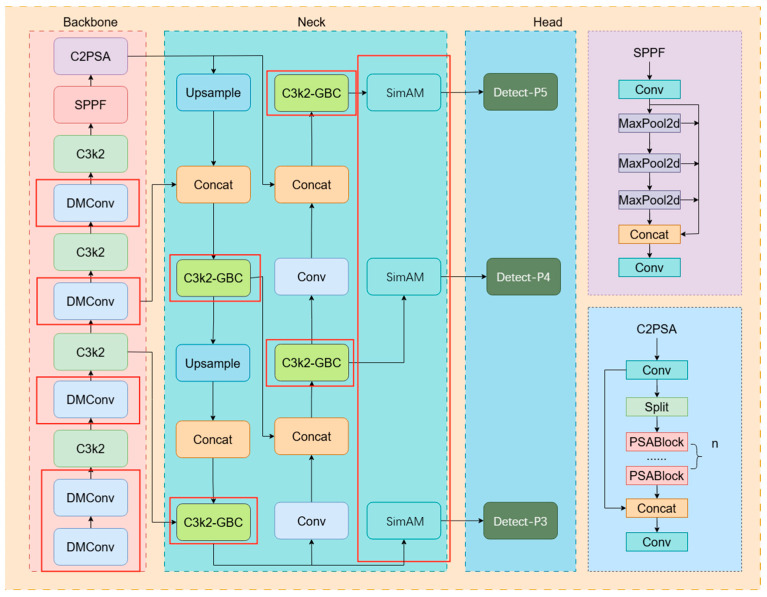
DGS-YOLO network model architecture.

When classifying pig facial images, YOLOv11n struggles to extract detailed features in complex backgrounds; at the same time, an excessive number of parameters also leads to prolonged model training and inference times, as well as high deployment costs. To address these issues, this paper proposes the DGS-YOLO model, which primarily employs the DMConv module to replace the Conv layer in the backbone for more effective extraction of texture detail features. Replacing C3K2 with C3K2-GBC in the neck layer enables the GBC module to capture multi-scale information while reducing the number of parameters and improving efficiency. In complex backgrounds, it achieves precise target recognition and better extraction of fine features, resolving issues caused by facial dirt or other objects partially obscuring the face. This facilitates more accurate target classification and effectively prevents overall model overfitting due to excessive parameters. By incorporating the SimAM attention mechanism before the detector head, the features undergo a final optimization to achieve optimal accuracy. Finally, regarding precision calculation, replacing the original loss function with the Shape-IoU loss function effectively addresses the inherent influence of bounding box shape and scale on IoU values in bounding box regression. This significantly enhances the experimental model’s generalization capability and accuracy. Through the synergistic interaction of multiple modules, the model resolves issues where complex backgrounds and excessive noise prevent accurate target locking, while also addressing occlusion problems in facial feature extraction. The improved DGS-YOLO model’s network architecture is shown in Figure 2. The red box indicates the improvements made in this model.

#### 2.2.2. DMConv

**Figure 3:**
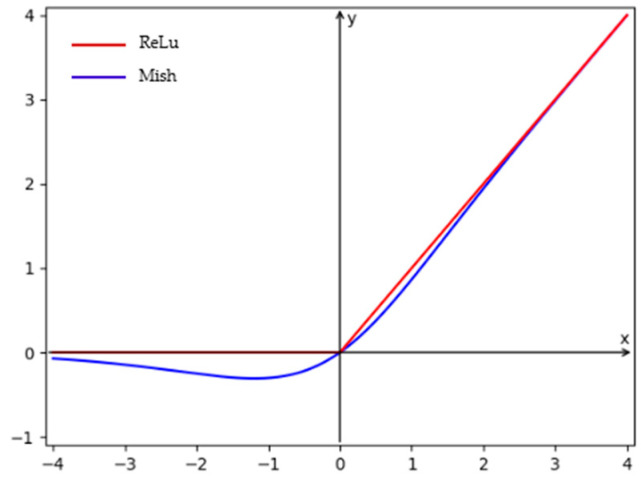
Graph of the Mish and Tanh functions.

DMConv is a convolutional module that combines the Mish activation function with dynamic normalization selection, primarily designed to enhance feature extraction capabilities through a smoother activation function and flexible regularization strategy. It retains the original DynamicTanh [10] structure and workflow while replacing the traditional Tanh function with the Mish [11] activation function to improve gradient flow and model expressiveness. The Mish and Tanh function graphs are shown in Figure 3.

**Figure 4:**
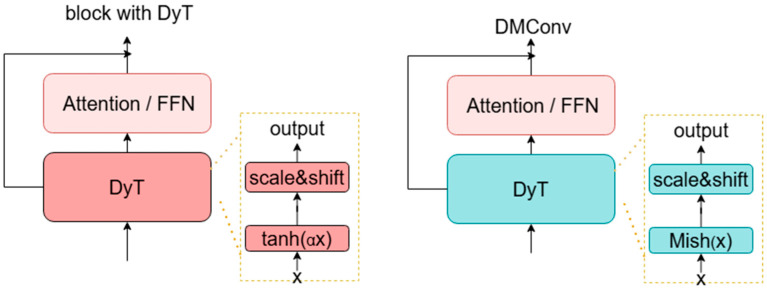
Schematic diagram of DynamicTanh and DMConv structures.

The primary workflow of DMConv involves performing convolution operations on raw features, extracting features, and performing channel transformations, followed by normalization processing to standardize feature distributions. Nonlinear transformations are then enhanced using the Mish activation function, with the final results output to the next layer. Modifications to the activation function reduce information loss during downsampling, preserve negative values to enrich low-level features such as edges and textures, effectively suppress noise interference in input images, and prevent gradient vanishing and explosion. The block diagrams of its DynamicTanh and DMConv are shown in Figure 4.

The Mish formula is defined as

$$f\left(x\right)=x\times \tanh\left(softplus\left(x\right)\right)=x\times tanh(ln(1+e^{x}))$$

DMConv and DynamicTanh were tested on the YOLOv11n dataset, with the experimental results shown in Table 1.

#### 2.2.3. GBC Model

**Figure 5:**
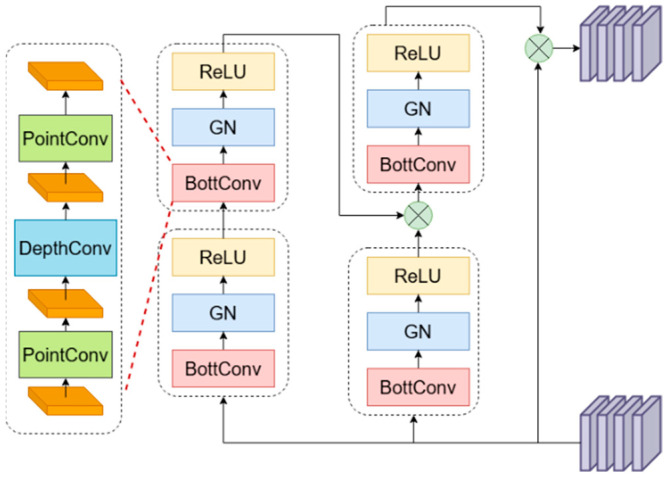
GBC architecture.

The gated bottleneck convolution (GBC) [12] module dynamically adjusts the weights of complex backgrounds and multi-morphological features to generate adaptive features for each spatial location and channel, thereby effectively enhancing the model’s ability to extract detailed information. This module employs a multi-path feature extraction and interaction mechanism. Each path incorporates bottleneck convolutions (BottConv), ReLU activation functions, and group normalization (GN). Serial 3 × 3 convolutions focus on local feature extraction, while parallel 1 × 1 convolutions facilitate inter-channel information exchange. These operations enhance key feature representations. Furthermore, GBC replaces traditional batch normalization (BN) with group normalization, making it more suitable for group convolution architectures and mini-batch training scenarios. Among these, bottleneck convolutions significantly reduce the number of parameters and computational overhead by mapping features from high-dimensional to low-dimensional spaces. In this model, GBC effectively captures multi-scale information. Its branching structure not only facilitates the interaction and enhancement of detailed features but also reduces model complexity and computational burden, thereby improving overall computational efficiency. The specific structure of GBC is illustrated in Figure 5.

First, as features pass through the convolutional layer, the number of channels doubles and is evenly split into two parts: one part is directly retained as the base features, while the other enters the feature extraction loop. Feature extraction occurs within the GBC module, which comprises four blocks. Features first pass through Block 1 and Block 2 (connected in series), each employing 3 × 3 convolutions. Selected modules sequentially process the features, with each module’s output feeding into the next. All intermediate features are retained throughout this process. After feature extraction, all features are concatenated along the channel dimension. Finally, the concatenated composite features undergo channel reduction via the cv2 convolution layer, adjusting the number of feature channels to the target output dimension, thus completing the entire forward propagation process. During forward propagation, another branch simultaneously undergoes feature extraction through Block 3. The results from both branches are then multiplied, implementing a dynamic gating mechanism that enhances key features while suppressing irrelevant information. The result undergoes final transformation through Block 4, concluding the feature extraction cycle. Residual connections are formed between the base features and the features extracted through the cycle, effectively merging original information with deeply refined information. This enhances the model’s ability to capture multi-scale details and contextual information in facial recognition.

#### 2.2.4. SimAM Attention Mechanism

SimAM [13] is a lightweight attention mechanism module that does not require any trainable parameters. Its core idea is based on the saliency detection theory in neuroscience, autonomously highlighting important features and suppressing irrelevant background interference by analyzing the statistical differences between each position in the feature map and its surrounding area. This module uses the mean and variance of feature maps across channels and spatial locations to construct an energy function, thereby quantifying the saliency of each location. Based on this energy value, SimAM can automatically assign differentiated weights to different positions, thereby enhancing feature responses relevant to the detection target without manual intervention while effectively suppressing interference from background noise. In the DGS-YOLO model of this study, feature information possesses rich semantic and location information after undergoing deep extraction through the backbone network and multi-level fusion via the neck network. Building upon this foundation, the introduction of the SimAM module prior to the detection head can be regarded as the final global optimization and refinement of the fused features. Through weight recalibration within this module, the model can further focus on regions most relevant to pig facial discrimination, thereby enhancing overall recognition accuracy and robustness.

#### 2.2.5. Shape-IoU Loss Function

In current pig face recognition research, object detection methods primarily rely on evaluation metrics such as IoU [14], GIoU [15], CIoU [16], and SIoU [17]. These approaches primarily determine bounding boxes through the geometric relationship between ground truth and predicted boxes and then compute losses based on the relative position and shape of the bounding boxes. However, they generally neglect the influence of inherent properties such as the shape and scale of the original bounding box on bounding box regression. Specifically, when regression sample bias and shape bias are identical and non-zero, and the ground truth (GT) bounding box exhibits differences in long and short sides, the dimensional discrepancy of the bounding box directly impacts the IoU value. Similarly, when the scale is identical but both regression samples and shape deviations are non-zero, the shape of the bounding box can also interfere with IoU results. If regression samples share the same non-zero shape deviation and the bounding box scale is small, the IoU value becomes more susceptible to the inherent characteristics of the ground truth (GT) box itself. Zhang et al. proposed Shape-IoU [18], which effectively addresses the aforementioned issues. Its specific formula is as follows:

(1) $$ww=\frac{{2\times \left(w^{gt}\right)}^{scale}}{{\left(w^{gt}\right)}^{scale}+{\left(h^{gt}\right)}^{scale}}$$

(2) $$hh=\frac{{2\times (h^{gt})}^{scale}}{{\left(w^{gt}\right)}^{scale}+{\left(h^{gt}\right)}^{scale}}$$

Here, scale represents the scale factor of the target in the dataset, *w* and *h* denote the width and height of the predicted bounding box, $w^{gt}$ and $h^{gt}$ denote the ground truth (GT) box width and height, respectively, and *ww* and *hh* denote the weighting coefficients for the horizontal and vertical directions, whose values are related to the shape of the actual bounding box.

(3) $${distance}^{shape}={hh\times \left(x_{c}-x_{c}^{gt}\right)}^{2}/c^{2}+{ww\times (y_{c}-y_{c}^{gt})}^{2}/c^{2}$$

(4) $$\Omega^{shape}=\sum_{t=w,h}{\left(1-e^{-\omega_{t}}\right)}^{\theta },\theta =4$$

(5) $$\left\{\begin{array}{c} \omega_{w}=hh\times \frac{\left|w-w^{gt}\right|}{max(w,w^{gt})}\\ \omega_{h}=ww\times \frac{\left|h-h^{gt}\right|}{max(h,h^{gt})} \end{array}\right.$$

The corresponding bounding box regression loss is as follows:

(6) $$L_{Shape-IoU}=1-IoU+{distance}^{shape}+0.5\times \Omega^{shape}$$

Regarding the impact of bounding boxes on the features and background issues of this dataset, the formula is defined as

(7) $$L_{Shape-IoU}=IoU-{distance}^{shape}-0.6\times \Omega^{shape}$$

**Figure 6:**
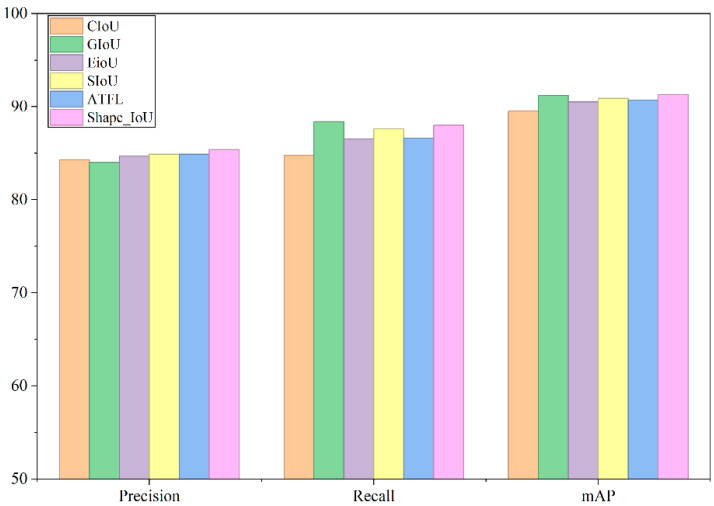
Comparison of loss functions in facial recognition.

To systematically evaluate the impact of different bounding box regression loss functions on the performance of pig facial recognition models, this study conducted comparative experiments using multiple mainstream loss functions within the unified YOLOv11n network architecture and this dataset. The experiments strictly controlled variables to ensure consistency in all training settings and evaluation criteria except for the loss function. The experimental results are shown in Table 2. For visual reference, see Figure 6. The results demonstrate strong performance in both accuracy and mAP50, with improvements of 1.1%, 3.2%, and 1.8% over the original CIoU, respectively.

## 3. Experimental Results and Analysis

### 3.1. Evaluation Criteria

In this study, precision, recall, and mAP50 are employed to evaluate facial recognition performance, with their respective formulas as follows:

(8) $$Precision=\frac{tp}{tp+fp}$$

(9) $$Recall=\frac{tp}{tp+fn}$$

(10) $$AP=\int_{0}^{1}P(k)dr$$

(11) $$mAP=\frac{1}{n}\sum_{k=1}^{k=n}AP_{k}$$

### 3.2. Experimental Details

To ensure experimental rigor, all experiments were conducted using identical equipment for training. Models were developed using the PyTorch deep learning framework within the Anaconda environment. The input image size was 640 × 640 pixels, with SGD as the optimizer. The initial learning rate was 0.01, the weight decay coefficient was 0.0005, the momentum was 0.937, the batch size was 32, the number of workers was 8, and the number of epochs was 180. The specific experimental environment configuration parameters are shown in Table 3.

### 3.3. Comparative Experiments of Different Models

**Figure 7:**
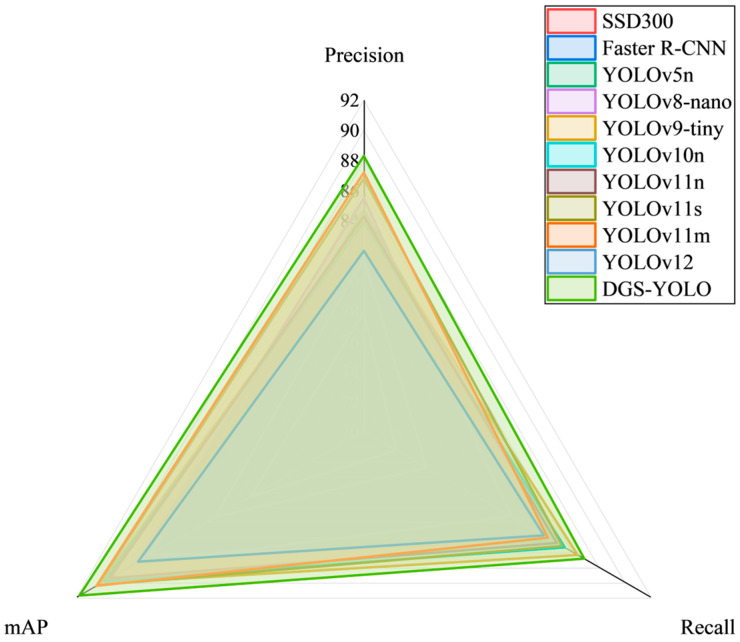
Comparison of experimental results.

To comprehensively validate the overall detection performance of the DGS-YOLO model in facial recognition of pigs under complex pig farming environments, and to ensure the reliability of the experimental process and the reproducibility of results, all experiments in this study were conducted using a unified dataset, hardware equipment, and software environment. We conducted a systematic performance evaluation of multiple mainstream detection models, focusing on comparative analysis of key metrics such as precision, recall, mAP50, and FLOPs. According to the experimental results shown in Table 4, although YOLOv11m achieved the highest recognition accuracy and mAP50 metrics, YOLOv11s generally outperformed YOLOv11n across all performance metrics. However, its higher FLOPs consumption limits its inference efficiency and practical deployment feasibility on resource-constrained edge devices. In contrast, the proposed DGS-YOLO model achieved significantly higher performance than the original YOLOv11n model across all three core metrics—precision, recall, and mAP50—while maintaining low computational complexity. It demonstrated improvements of 4%, 2.1%, and 2.3%, respectively, alongside a further reduction in FLOPs of 0.5 G, reflecting superior overall performance. After conducting a comprehensive comparison of multiple mainstream detection networks, we ultimately selected YOLOv11n as the foundational architecture for enhancement, taking into account multiple factors including model accuracy, recall rate, mAP50, and computational efficiency. Building upon this model, we introduced several structural optimization strategies. As a result, the proposed DGS-YOLO model achieved an optimal balance across multiple evaluation metrics, ensuring high recognition accuracy while demonstrating superior practical deployment applicability. Detailed experimental results are compared in Figure 7.

### 3.4. Ablation Experiment

On the specialized dataset constructed in this paper, we systematically conducted a series of ablation experiments to individually validate the specific contributions of each proposed improvement module to the model’s performance. These experiments used the standard YOLOv11 network as the baseline model and progressively introduced the following three key improvement structures for comparative analysis: First, we adopted a Dynamic Detail-Enhancing Convolution (DMConv) module in the backbone network. Second, we replaced the C3k2 structure in the neck network with a C3k2-GBC module featuring gated and bottleneck optimization design. Third, we introduced a parameter-free SimAM attention mechanism at the detection head front end. The experimental results demonstrate that introducing the DMConv structure into the YOLOv11n backbone network alone improved model recognition accuracy from the baseline of 84.30% to 85.5%, indicating that this module plays a significant role in capturing complex textures and subtle features on pig faces. Furthermore, when the C3k2-GBC module was used alone to replace the original neck structure, it outperformed the original model in three out of four core evaluation metrics. Specifically, accuracy reached 86.7%, mAP50 improved to 90.6%, and FLOPs decreased to 5.9 G, demonstrating the module’s advantages in feature fusion and computational efficiency, effectively resolving the issue of accuracy being compromised due to obstruction problems. When SimAM attention mechanism was introduced independently, there was no improvement in accuracy, recall, or mAP50. However, through multiple experiments, it was found that the coordinated use of SimAM with other multi-module components enabled the model to achieve the highest accuracy. It is worth noting that the C3k2-GBC module achieved outstanding accuracy when used independently, primarily due to its gated bottleneck convolution (GBC) structure. This structure dynamically adjusts the weight distribution between background information and fine-grained features. The embedded BottConv unit optimizes redundant information while preserving critical details, thereby enhancing the model’s ability to extract fine-grained features in complex scenes.

Ultimately, we integrated the three effective structures mentioned above to construct a complete DGS-YOLO model, which was trained using a shape-adaptive Shape-IoU loss function. Experiments demonstrated that this fusion model achieved optimal performance across multiple key metrics, including accuracy, recall, and mAP50. By introducing the parameter-free SimAM attention mechanism and lightweight DMConv structure, the model achieved a slight reduction in total parameters. Simultaneously, thanks to C3k2-GBC optimization of the neck network, overall model complexity was further reduced, ultimately maintaining FLOPs at 5.9 G. Specifically, compared to the original YOLOv11n network, DGS-YOLO achieved a 4% improvement in accuracy, a 2.1% increase in recall, and a 2.3% boost in mAP50. Comprehensive experimental results confirm that all innovative modules introduced in DGS-YOLO demonstrate clear effectiveness, providing an enhanced deep learning solution for pig facial recognition tasks in complex environments. The ablation experiment results are shown in Table 5. The checkmark (√) in the table indicates that a specific feature or augmentation was included in the evaluated model configuration.

### 3.5. Model Small-Sample Testing Experiment

**Figure 8:**
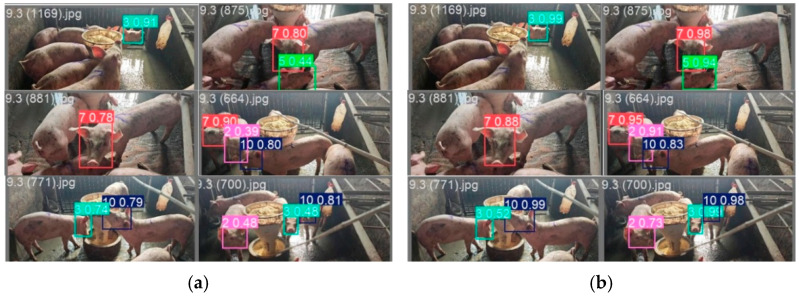
(**a**) The visualization of the YOLOv11n model experiment. (**b**) The visualization of the DGS-YOLO model experiment.

In this study, we conducted comparative experiments between YOLOv11n and the DGS-YOLO model proposed in this paper on a small-sample pig face dataset. This dataset contained a total of 938 images across eight categories. The training set comprised 750 images, while the test set consisted of 188 images. The detailed experimental results are shown in Table 6. It is evident that DGS-YOLO achieved significant improvements over the original YOLOv11n across key performance metrics: detection accuracy increased by 20.2%, and the mAP50 metric improved by 10.3%. The experimental results are shown in Figure 8. Six randomly selected image results are displayed, with the left image showing the recognition results from the YOLOv11n baseline model and the right image showing the recognition results from our DGS-YOLO model. For example, regarding the recognition accuracy rates for labels “2”, “3”, and “10” in the 9.3 (700) jpg image, the original YOLOv11n achieved accuracies of 0.48, 0.48, and 0.81, respectively. In contrast, our model achieved accuracies of 0.73, 0.99, and 0.98. This demonstrates that our model maintains strong performance on small datasets even under conditions of severe occlusion and complex backgrounds. This comparative analysis conclusively demonstrates that DGS-YOLO, through structural optimization and enhanced feature extraction mechanisms, effectively improves its adaptability to small-sample datasets and its ability to represent features. Based on the experimental results obtained from conventional-sized datasets, it can be concluded that this model not only performs exceptionally well under abundant data conditions but also demonstrates strong generalization capabilities and robustness when confronted with limited training samples. This indicates its significant practical value and application potential, particularly for animal individual recognition tasks in the field of intelligent agriculture where data acquisition costs are high.

### 3.6. Facial Recognition Analysis

**Figure 9:**
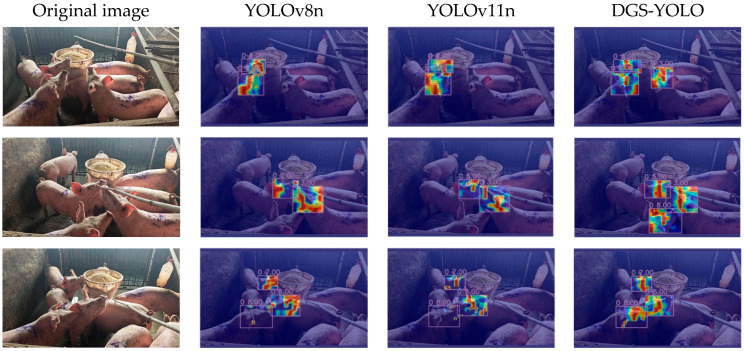

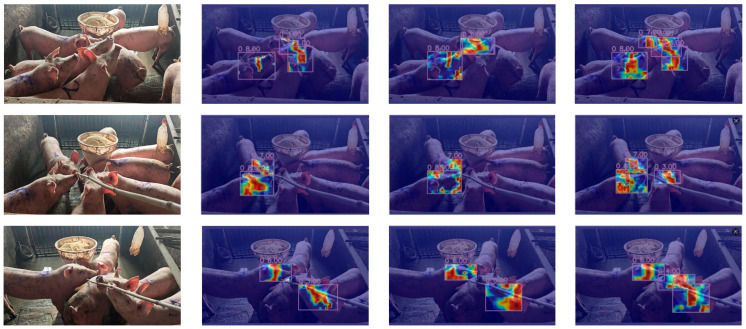
Grad-CAM facial visualization.

To enable comparison with other models, this paper employed Gradient-Weighted Class Activation Maps (Grad-CAM) [24] to visualize facial data processing outputs for YOLOv8n, YOLOv11n, and DGS-YOLO. As shown in the occlusion case depicted in the first image of Figure 9, compared to the missed detections observed in the YOLOv8n and YOLOv11n baseline models, the proposed model achieved more complete recognition, accurately detecting all three pigs’ faces. Additionally, as clearly demonstrated by the remaining images, other models tended to misidentify background elements as facial features or fail to fully recognize facial features. In contrast, our model achieved more precise facial feature recognition, maintaining accuracy even under severe occlusion conditions. The results demonstrate that our model achieves more precise extraction of facial features in complex backgrounds, showcasing its robust performance. The experimental results are shown in Figure 9.

### 3.7. Generalization Experiment

**Figure 10:**
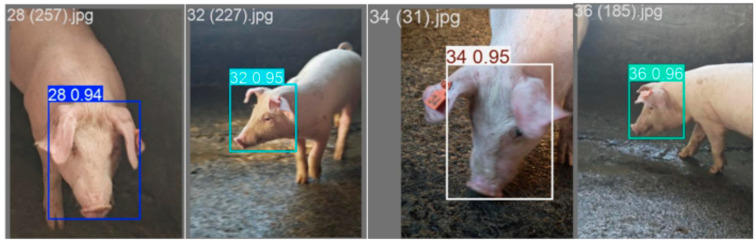
Generalization experiment results.

To validate the robustness of this model, we conducted generalization experiments on pigs of multiple age groups across different environmental piggeries. The images used in this experiment were captured from approximately one-month-old piglets raised during winter, yielding a total of 736 facial images through single-angle photography. The dataset was split into training and validation sets at an 8:2 ratio. Labelimg was used to annotate bounding boxes around pig faces in the images, with label categories “28, 32, 34, 36”. Facial recognition tests using this model yielded the results shown in Figure 10. The findings demonstrate that the model maintains strong recognition performance for young piglets across different rearing environments, reflecting robust environmental adaptability and generalization capabilities.

## 4. Discussion

Against the backdrop of increasingly widespread intelligent livestock management, this paper proposes an optimized DGS-YOLO network model based on the widely adopted deep learning object detection framework YOLOv11n. This approach aims to enhance the accuracy and efficiency of facial recognition for groups of pigs, thereby further serving practical applications such as disease prevention and control, as well as agricultural insurance claims processing. This model implements multi-level structural improvements to address several critical challenges in pig facial recognition tasks: First, the standard convolutional layers (Conv) in YOLOv11n are replaced with detail-enhancing convolutional layers (DMConv) to enhance the extraction of subtle facial features in pigs. Secondly, introducing the C3k2-GBC module into the neck network to replace the original C3k2 architecture not only effectively reduces model complexity but also enhances target localization accuracy. This partially mitigates recognition interference caused by factors such as facial blemishes and occlusions. Furthermore, this study incorporates the SimAM attention mechanism to optimize fused features, thereby suppressing the negative impact of complex backgrounds on recognition results. Simultaneously employing the Shape-IoU loss function enables more precise regression of bounding boxes, thereby significantly improving detection accuracy in experiments. Experimental results demonstrate that DGS-YOLO exhibits strong recognition performance on the dataset constructed in this study and in scenarios with small sample sizes. However, when confronted with certain external samples, its detection accuracy remains slightly lower than that of some existing state-of-the-art models. Therefore, in subsequent research, differentiated feature extraction strategies can be designed based on the specific characteristics of different pig herd samples. Targeted optimization of the model architecture can be performed to enhance its cross-scenario adaptability and recognition robustness. Additionally, the model can enhance cross-platform deployment capabilities on edge devices while improving detection accuracy and inference speed by refining the design of detail feature extraction layers or incorporating more lightweight network modules. This better meets the real-time, efficient recognition demands of actual farming environments.

## 5. Conclusions

The DGS-YOLO model proposed in this paper implements several key structural optimizations based on YOLOv11n. Through systematic experimentation, the following main conclusions were drawn:

1. Compared to the original YOLOv11n model, the proposed DGS-YOLO achieves significant improvements across multiple core evaluation metrics, including detection accuracy, recall, and mean average precision (mAP50). Although ablation studies reveal that certain standalone improvement modules may cause temporary fluctuations in some metrics, the model achieves optimal overall performance when all optimization strategies are applied collectively, demonstrating effective synergistic interactions among the modules.
2. In comparative experiments against other mainstream object detection models, DGS-YOLO demonstrated significant advantages. It outperformed SSD and Faster-RCNN models by approximately 10% in detection accuracy, superior to most competing models. Additionally, the network achieved a slight reduction in parameter count, effectively controlling overall model complexity. This facilitates deployment in real-world environments and helps address challenges in applying models within resource-constrained scenarios.
3. In addressing key challenges encountered in real-world farming scenarios, DGS-YOLO demonstrates strong robustness. The model effectively handles facial recognition difficulties caused by occlusions, enhancing its ability to discern local features through deep detail feature extraction mechanisms. Simultaneously, in detection tasks with limited sample sizes, the model exhibits superior generalization performance and stability compared to the original YOLOv11n, indicating promising practical applications.

## Figures and Tables

**Table 1:** Evaluation metrics for DynamicTanh and improved DMConv in this experiment.

Add Module Name	Precision	Recall	mAP50
DynamicTanh	0.844	0.847	0.882
DMConv	0.857	0.84	0.887

**Table 2:** Shape-IoU values compared with other models.

Name	Precision	Recall	mAP50
CIoU	0.843	0.848	0.895
GIoU	0.840	0.884	0.912
EIoU [19]	0.847	0.865	0.905
SIoU	0.849	0.876	0.909
ATFL [20]	0.849	0.866	0.907
Shape-IoU	0.854	0.880	0.913

**Table 3:** Environment configuration parameters.

Environment Configuration	Parameter
CPU	Intel(R) Core(TM) i9-10920X CPU @ 3.50 GHz
GPU	NVIDIA GeForce RTX 3080
Development environment	PyCharm 2023.2.5
Language	Python 3.8.18
Framework	PyTorch 2.0.1
Operating platform	CUDA 11.8

**Table 4:** Comparison with other models.

Name	Precision	Recall	mAP50	FLOPs (G)
SSD300	0.779	0.725	0.787	30.8
Faster R-CNN	0.815	0.748	0.814	37.6
YOLOv5n	0.847	0.845	0.865	7.1
YOLOv8-nano	0.855	0.813	0.876	8.1
YOLOv9-tiny [21]	0.841	0.864	0.902	7.7
YOLOv10n [22]	0.839	0.854	0.899	8.3
YOLOv11n	0.843	0.848	0.895	6.4
YOLOv11s	0.868	0.852	0.904	21.5
YOLOv11m	0.872	0.841	0.905	68.1
YOLOv12 [23]	0.82	0.838	0.873	6.5
DGS-YOLO	0.883	0.869	0.918	5.9

**Table 5:** Ablation experiments.

	DMConv	C3K2-GBC	SimAM	Shape-IOU	Precision	Recall	mAP50	FLOPs
1					0.843	0.848	0.895	6.4
2	√				0.855	0.84	0.887	6.4
3		√			0.867	0.84	0.906	5.9
4			√		0.842	0.848	0.895	6.4
5	√	√			0.869	0.844	0.903	5.9
6		√	√		0.857	0.848	0.898	5.9
7	√		√		0.847	0.86	0.896	6.4
8	√	√	√		0.878	0.854	0.911	5.9
Ours	√	√	√	√	0.883	0.869	0.918	5.9

**Table 6:** Small-sample comparative experiment.

Name	Precision	Recall	mAP50
YOLOv11n	0.561	0.744	0.705
DGS-YOLO	0.763	0.747	0.808

## Data Availability

The original contributions presented in this study are included in the article. Further inquiries can be directed to the corresponding authors.
